# Hypoxia‐preconditioned MiotEVs from bone marrow mesenchymal stem cells inhibit myocardial infarction‐induced cardiac fibrosis

**DOI:** 10.1002/btm2.70046

**Published:** 2025-07-04

**Authors:** Jungang Nie, Hongwen Zhu, Zhiming Gao, Liang Wang

**Affiliations:** ^1^ Department of Cardiology The First Affiliated Hospital of Nanchang University Nanchang Jiangxi China; ^2^ Hypertension Research Institute of Jiangxi Province Nanchang Jiangxi China

**Keywords:** cardiac fibrosis, energy metabolism, glycolysis, QKI, MitoEVs, myocardial infarction

## Abstract

Hypoxia‐preconditioned bone marrow mesenchymal stem cell‐derived mitochondrial extracellular vesicles (Hypoxia‐BMSC MitoEVs) emerged as a novel therapeutic candidate for myocardial infarction (MI)‐induced cardiac fibrosis. Here, we demonstrate that MitoEVs isolated from hypoxic BMSCs, rich in intact mitochondria and the RNA‐binding protein Quaking (QKI), potently inhibited TGF‐*β*1‐driven myofibroblast activation in vitro by suppressing *α*‐SMA and collagen expression while restoring mitochondrial oxidative phosphorylation and metabolic balance. In a murine MI model, systemic delivery of Hypoxia‐BMSC MitoEVs attenuated cardiac fibrosis, reduced infarct size, and improved left ventricular function. Pharmacological inhibition of mitochondrial ATP synthase in MitoEVs similarly diminished their therapeutic efficacy. Mechanistically, MitoEVs delivered QKI protein to cardiac fibroblasts, where it inhibited translation of fibrotic mRNAs via m7G‐modified RNA interactions. Genetic ablation of QKI in BMSCs abrogated MitoEV‐mediated antifibrotic effects both in vitro and in vivo, confirming QKI as a critical effector. These results suggested that both QKI‐driven translational suppression and mitochondrial bioenergetics underpin their antifibrotic action. These findings highlight Hypoxia‐BMSC MitoEVs as a therapeutic strategy to mitigate post‐MI fibrosis, warranting further exploration for clinical translation.

**Translational Impact Statement**

This study identifies hypoxia‐preconditioned mitochondrial extracellular vesicles (Hypoxia‐BMSC MitoEVs) as a novel dual‐action therapy to combat post‐myocardial infarction (MI) cardiac fibrosis. MitoEVs deliver functional mitochondria to restore cardiac bioenergetics and transfer the RNA‐binding protein QKI to suppress fibrotic mRNA translation, synergistically targeting metabolic dysfunction and pathological fibroblast activation. These findings advance MitoEV‐based therapies toward clinical testing for post‐MI remodeling, offering a multitargeted approach beyond current antifibrotic strategies limited to extracellular matrix modulation.

## 1 INTRODUCTION

Cardiac fibrosis is a pathological condition characterized by the excessive accumulation of fibrous connective tissue in the heart.^1^ It is a common feature of various cardiovascular diseases, including myocardial infarction (MI), hypertension, and cardiomyopathies. The normal healing process after cardiac injury involves the activation of cardiac fibroblasts (CF), which produce extracellular matrix (ECM) proteins to repair damaged tissue.^2^ However, in pathological conditions, such as chronic inflammation or prolonged mechanical stress, this reparative process becomes dysregulated, leading to excessive deposition of ECM components, primarily collagen.^3^ This disrupts the normal architecture and function of the heart, impairing its ability to contract effectively.^4^ Cardiac fibrosis has several detrimental effects on cardiac function. The increased stiffness of the fibrotic tissue impairs myocardial relaxation, limiting the heart's diastolic filling capacity.^5^ This results in an increased diastolic pressure, leading to symptoms such as shortness of breath and fatigue.^6^ Additionally, the altered ECM composition and structure disrupt the electrical conduction system of the heart, contributing to arrhythmias and conduction abnormalities.^7^ Furthermore, cardiac fibrosis promotes the development of a pro‐inflammatory and pro‐fibrotic microenvironment, perpetuating a vicious cycle of fibrosis progression.^8^ This microenvironment stimulates the activation of CFs, further enhancing ECM production and exacerbating fibrosis. Recent research has highlighted the involvement of various signaling pathways and molecular mechanisms in the development and progression of cardiac fibrosis.^9^ Transforming growth factor‐beta (TGF‐*β*), angiotensin II, and reactive oxygen species (ROS) are key mediators implicated in fibrotic signaling cascades.^10^ These signaling molecules activate intracellular pathways that promote fibroblast activation, differentiation into myofibroblasts, and ECM synthesis.^11^ Understanding the underlying mechanisms and cellular interactions involved in cardiac fibrosis is crucial for the development of effective therapeutic strategies.

MitoEVs, or mitochondrial‐derived extracellular vesicles, have emerged as a fascinating area of study within the field of extracellular vesicle (EV) research.^12^ These vesicles are derived from mitochondria, which are known as the powerhouses of the cell responsible for generating energy and regulating various cellular processes.^13^ EVs are small membrane‐bound vesicles released by cells into the extracellular space.^14^ They play crucial roles in intercellular communication by transferring various bioactive molecules, including proteins, lipids, and nucleic acids, between cells.^15^ EVs have been extensively studied for their roles in physiological processes as well as in disease pathogenesis. MitoEVs, a specific subpopulation of EVs, are distinct in their origin and cargo as they originate directly from mitochondria.^16^ They contain not only mitochondrial proteins but also mitochondrial nucleic acids, including mtDNA, mtRNA, and mt‐miRNAs. These unique components make MitoEVs attractive targets for investigating their potential roles in cellular homeostasis, disease progression, and therapeutic applications.^17^ Research has shown that MitoEVs play important roles in intercellular communication and can influence various biological processes.^18^ They have been implicated in modulating cellular metabolism, immune responses, oxidative stress, and apoptosis. Additionally, MitoEVs have been found to participate in the transfer of damaged mitochondria and mitochondrial components for degradation, contributing to cellular quality control mechanisms.^19^ The release and uptake of MitoEVs can be regulated by both physiological and pathological conditions. Various stimuli, such as mitochondrial dysfunction, oxidative stress, or inflammation, can trigger the release of MitoEVs from cells. Once released, these vesicles can be taken up by neighboring or distant cells, delivering their cargo and potentially influencing cellular functions and responses.^20^, ^21^ Understanding the functions and mechanisms of MitoEVs is a rapidly evolving area of research with implications for a wide range of fields, including cell biology, physiology, and disease pathogenesis. The identification and characterization of MitoEVs hold great potential in unraveling novel intercellular communication pathways and discovering new biomarkers and therapeutic targets for mitochondrial‐related diseases, such as neurodegenerative disorders, cardiovascular diseases, and cancer.

BMSCs, or bone marrow‐derived mesenchymal stem cells, are a type of multipotent stem cell found in the bone marrow.^19^ They have gained significant attention and recognition in regenerative medicine and tissue engineering due to their unique properties and potential therapeutic applications. BMSCs are characterized by their ability to self‐renew and differentiate into multiple cell types, including osteoblasts (bone‐forming cells), chondrocytes (cartilage‐forming cells), adipocytes (fat cells), and other mesenchymal lineages.^22^ This multilineage differentiation potential makes BMSCs a valuable resource for tissue repair and regeneration. Bone marrow is a rich source of BMSCs, and they can be easily isolated through minimally invasive procedures such as bone marrow aspiration.^23^ Once isolated, BMSCs can be expanded in culture while maintaining their stem cell characteristics. These cells have the capacity to proliferate extensively, providing many cells for various therapeutic applications.

Hypoxia, or low oxygen levels, is a physiological condition that occurs in various tissues and organs during normal development and under certain pathological conditions.^24^ It has been observed that hypoxia can significantly influence cellular behavior and function. In the case of BMSCs, exposure to hypoxia triggers a series of adaptive responses that can enhance their therapeutic properties. During hypoxic preconditioning, BMSCs experience changes in gene expression and cellular metabolism, leading to the secretion of specific factors and enhancement of certain cellular functions. For example, hypoxia induces the release of growth factors, cytokines, and chemokines that promote tissue repair, angiogenesis (formation of new blood vessels), and immunomodulation.^25^ Hypoxia preconditioned BMSCs have shown promising results in preclinical and clinical studies across various fields, including cardiovascular diseases, neurological disorders, musculoskeletal injuries, and wound healing. Their enhanced therapeutic properties make them more effective in promoting tissue repair, improving functional recovery, and modulating immune responses.

In this study, we investigate a novel therapeutic approach for addressing cardiac fibrosis. The research focuses specifically on the use of hypoxia‐preconditioned MiotEVs derived from BMSCs. The study provides novel insights into the potential therapeutic application of hypoxia‐preconditioned MiotEVs for cardiac fibrosis. By harnessing the regenerative potential of bone marrow mesenchymal stem cells (BMSCs) and their derived MiotEVs, this approach offers a promising strategy to combat the detrimental effects of cardiac fibrosis following MI. The results highlight the value of utilizing hypoxic preconditioning to enhance the therapeutic properties of stem cell‐derived EVs.

## 2 MATERIALS AND METHODS

### 2.1 Culture and characterization of bone marrow MSCs (BMSCs)

Cyagen Biosciences (Guangzhou, China) provided adult BMSCs, which were then cultured in a complete growth medium consisting of DMEM/F‐12 medium supplemented with 10% FBS and 1% P/S. The cells were incubated at 37°C in a 5% CO_2_ cell culture incubator, and once they reached 70%–80% confluence, the adherent cells were passaged. Subsequent experiments utilized P4 cells. To characterize the phenotype, BMSCs were labeled with polyclonal antibodies against CD14, CD19, CD45, CD90, CD105, and CD73 (BD Pharmingen, San Diego, CA, USA) and examined using flow cytometry (BD Biosciences, Franklin Lakes, NJ, USA). FlowJo™ V10 software was utilized for the analysis of the data. To analyze the potential for differentiation in multiple directions, we utilized osteogenic induction medium, adipogenic induction medium, chondrogenic induction medium, and neuron induction medium (all sourced from Cyagen, Suzhou, China) to substitute the complete medium and initiate the process of differentiation. The manufacturer's instructions were followed to induce differentiation, and the assessment was conducted using Alizarin red staining, Oil Red O staining, and TRAP staining.

### 2.2 Preparation and identification of MitoEVs

The process of preparing and identifying MitoEVs was carried out according to a previously published method. Supplementary Figure 1 displayed the process. Bone marrow stromal cells (BMSCs) were cultured in regular oxygen cell incubators at a temperature of 37°C with 5% carbon dioxide and 21% oxygen, or in hypoxic cell incubators with 5% oxygen in DMEM/F12 medium containing 10% EV‐depleted fetal bovine serum (Vivacell, Shanghai, China) for a duration of 48 h. Afterward, the medium was gathered and subjected to centrifugation at 3000 times the force of gravity for 10 min to eliminate deceased cells, followed by another centrifugation at 10,000 times the force of gravity for 30 min to eliminate cellular debris. The liquid above the sediment was subjected to ultracentrifugation at a speed of 120,000 times the force of gravity at a temperature of 4°C for a duration of 1 h, repeated twice. The small balls were mixed again in a solution of phosphate‐buffered saline (PBS). MitoEVs were either utilized promptly or preserved at a temperature of −80°C. The concentration and size distribution of MitoEVs were measured using NanoSight tracking analysis (NTA, Malvern, UK). The morphology of MitoEVs was identified using transmission electron microscopy (TEM) from Joel in Tokyo, Japan. For the Western Blotting analysis of surface markers and mitochondria, proteins were separated on 10% SDS‐PAGE gels, transferred to PVDF membranes, and probed with antibodies against Alix1 (1:1000, Abcam ab186429), CD81 (1:1000, Santa Cruz sc‐166029), TSG101 (1:1000, Abcam ab125011), cytochrome c (1:1000, Cell Signaling 11940), and calnexin (1:1000, Abcam ab22595). The EVs were labeled with PKH67 (PKH67GL‐1KT, Sigma, GER Burlington, MA, USA) using the instructions provided by the manufacturer. After incubating the labeled MitoEVs with CFs for 12 h, the fibroblasts were fixed using 4% paraformaldehyde. Then, they were mounted with a DAPI‐containing mounting medium (Abcam, USA) and examined using a laser confocal microscope (Zeiss, LSM800, Germany).

### 2.3 Adult mouse CF isolation and immunofluorescence staining

Water was used to wash the excised and minced hearts of euthanized adult mice. Next, a mixture for digestion was prepared by adding 0.25% trypsin and 5 mg/mL Liberase TL to the minced tissue. The digestion process was carried out at 37°C for 30 min with continuous stirring. After centrifuging the mixture at a speed of 1500 rpm for a duration of 5 min, the obtained cell pellet was suspended again in fibroblast growth media. Afterward, the cells were placed onto plates coated with gelatin and kept at a temperature of 37°C for incubation. Following a 24‐h period, cells that did not adhere were removed, while the cells that adhered were placed in fibroblast growth media for further cultivation. For subsequent experiments, fibroblasts from either passage 1 or 2 were utilized. In order to trigger the differentiation of myofibroblasts, the culture media was supplemented with 10 ng/mL of TGF*β*1 for a duration of 24 h prior to harvesting. Specific primary antibodies were used for immunofluorescence staining and incubated overnight at 4°C. Following the washing process, the cells were exposed to the suitable secondary antibody for a duration of 2 h at ambient temperature. In the end, the discolored cells were placed on a slide with a mounting medium that contained DAPI and were then observed using a Zeiss LSM800 confocal microscope.

### 2.4 Cell viability assay

The proliferation of cells was evaluated by employing the Cell Counting Kit‐8 (Dojindo, Kumamoto, Japan) in accordance with the guidelines provided by the manufacturer. In short, a 96‐well plate was used to seed CFs (1 × 10^4^ cells/well), which were then subjected to treatment with TGF‐*β* for a duration of 72 h. Following a 4‐h incubation period with 10 μL of CCK‐8 solution, an analysis was conducted on the cells in every well. Using a Synergy H1 hybrid multimode microplate reader (BioTek, Winooski, VT, USA), the measurement of absorbance at 450 nm was conducted.

### 2.5 RNA isolation and qPCR

TriZol reagent, acquired from Thermo Fisher Scientific (Waltham, MA, USA), was used to extract RNA from samples of murine cardiac tissue and fibroblasts. To extract the RNA, chloroform, isopropanol, and ethanol were utilized in the protocol. To perform real‐time qPCR, 1 μg of RNA was utilized as the input for reverse transcription‐quantitative polymerase chain reaction. To conduct the qPCR analysis, the SYBR Green system for quantitative polymerase chain reaction (qPCR) was utilized, which is also provided by Thermo Fisher. For the quantification of each mRNA, specific primers were employed, and their sequences are available in Supplemental Table 1. The normalization of mRNA quantification was based on the expression of the housekeeping gene GAPDH. Comparing the mRNA levels of the target gene to the reference gene, the results were expressed as fold change values (2^−ΔΔ*Ct*^).

### 2.6 Western blotting

To extract lysates from murine heart tissues or cells, the subsequent procedures were executed. The tissues or cells underwent digestion in a lysis buffer (1X) comprising protease and phosphatase inhibitors, along with PMSF (Sangon Biotech) added. The digestion procedure was performed utilizing a Qiagen Tissue Lyser under 4°C conditions for a duration of 8 min at a speed of 50 rpm. Following the process of digestion, the lysates were gently agitated for a duration of 1 h at a temperature of 4°C. Subsequently, the cells were subjected to sonication in order to enhance their disruption and facilitate the release of the proteins. The lysates were quantified for protein concentration using the PierceTM BCA protein assay kit provided by Thermo Fisher. In SDS‐PAGE electrophoresis, a 10% polyacrylamide gel was used to load 20 μg of protein from every lysate sample. The gels from SDS‐PAGE were moved onto PVDF membranes (Millipore, Billerica, MA, USA) and subsequently obstructed with 5% BSA (Sigma‐Aldrich, CA, USA) for 1 h at a temperature of 25°C in the room. The membrane was incubated with the primary antibody at a concentration of 1:1000, while rocking for 1 h at a temperature of 4°C. After incubating the primary antibody, the membrane was treated with secondary antibodies conjugated with HRP and left to incubate for 1 h at room temperature with rocking. The blots were prepared with a Tanon High‐signature ECL Western Blotting Substrate from Shanghai, China. The following procedures facilitated the creation of lysates, measurement of protein concentration, segregation of proteins through SDS‐PAGE, transfer of proteins onto a PVDF membrane, incubation with antibodies, and the ultimate generation of Western blot signals utilizing an ECL substrate.

### 2.7 MI‐induced cardiac fibrosis model

All animal protocols were approved by the Nanchang University Laboratory Animal Research Committee and complied with ARRIVE guidelines; 8–10‐week‐old male BALB/c mice (Shanghai Laboratory Animal Center) were housed under SPF conditions, stratified by weight, and randomly assigned (*n* = 6/group) to Sham (thoracotomy without ligation, PBS i.v. daily for 5 days post‐surgery), MI + Vehicle (LAD ligation + PBS i.v. daily for 5 days post LAD), MI + Normoxic MitoEVs (LAD ligation + normoxia‐derived MitoEVs, 100 μg/kg i.v. daily for 5 days post LAD), or MI + Hypoxic MitoEVs (LAD ligation + hypoxia‐preconditioned MitoEVs, 100 μg/kg i.v. daily for 5 days post LAD) groups using blinded randomization; surgeries were performed under pentobarbital anesthesia (50 mg/kg i.p.) with LAD ligation confirmed by ECG and pallor, and MitoEVs were administered 24 h post‐MI. For the inhibition of ATP synthase, oligomycin (5 μg/mL) was used to treat the Hypoxia‐MitoEVs. For the QKI knockdown, BMSCs were transduced with lentiviral shQKI (Genelily, Shanghai, China) or scrambled control (shNC). Puromycin (2 μg/mL) was used for selection. QKI knockdown efficiency was confirmed by Western blot (QKI antibody: 1:1000, Abcam ab236538). Hearts were harvested 4 weeks post‐MI after CO_2_ euthanasia, with inclusion criteria requiring survival >48 h and successful ligation, and blinding during histological/molecular analyses; data were analyzed by one‐way ANOVA/Tukey's test.

### 2.8 Masson staining

Heart samples were fixed in 4% Paraformaldehyde (PFA) acquired from Sangon Biotech (Shanghai, China) to evaluate the morphological alterations and degree of cardiac fibrosis. Subsequently, the samples were embedded in paraffin. The assessment of cardiac fibrosis was conducted by employing Masson's Trichrome staining kit acquired from Solarbio (Beijing, China). Heart cross‐sections, embedded in paraffin and with a thickness of 5 μm, underwent staining using the Masson's Trichrome staining procedure. For subsequent analysis, pictures were taken using a light microscope at a 200× magnification. The quantification of the fibrotic portion was performed with Image J software from the National Institutes of Health in Bethesda, MD, USA. This was achieved by dividing the area of blue (representing fibrosis) by the overall area of the myocardium.

### 2.9 Seahorse analysis

In order to examine the bioenergetics of mitochondria in cellular samples, we employed the Seahorse XF24 device manufactured by Agilent Technologies, located in Santa Clara, CA. The procedure was carried out in the following manner: Cells (104/well) were placed in XF24‐well culture plates and given 24 h to attach. Following the experimental design, the cells underwent subsequent treatment. Prior to the analysis, the cells were rinsed two times with XF basic solution lacking sodium bicarbonate, which was then balanced to pH 7.4. Additionally, the cells were provided with 10 mM glucose and placed in a non‐CO_2_ incubator at a temperature of 37°C for a duration of 1 h. The Seahorse XF24 Analyzer was used to measure the oxygen consumption rate (OCR) and extracellular acidification rate (ECAR) during the assay. This was done by sequentially injecting compounds that impact oxidative phosphorylation (oligomycin, carbonyl cyanide‐4 (trifluoromethoxy) phenylhydrazone (FCCP), and antimycin A/rotenone). The analysis of the data was performed using Wave software from Agilent Technologies.

### 2.10 Tryptic digestion and LC/MS analysis

To prepare the MS samples, the proteins (100 μg) obtained from the MitoEVs were initially diminished in 20 mM dithiothreitol (Sigma Alderich, USA) at a temperature of 56°C for a duration of 2 h. Following that, they were treated with 40 mM iodoacetamide (Sigma Alderich, USA) for 30 min in the absence of light at ambient temperature to accomplish alkylation. After alkylation, the samples were moved to a 10 kDa centrifugal spin filter from Sartorius AG in Germany. They were then washed three times with 300 μL of 8 M urea and two times with 300 μL of 20 mM ammonium bicarbonate through centrifugation at 14,000 g. Following that, trypsin digestion was carried out by adding trypsin from Promega in the USA at a ratio of 1:50 (enzyme/substrate, m/m) in 200 μL of 20 mM ammonium bicarbonate at 37°C for 16 h. The peptides were collected by transferring the filter to a new collection tube and spinning at 14,000 g. To ensure complete peptide recovery, the filter was rinsed twice with 100 μL of 20 mM ammonium bicarbonate. After trypsin treatment, the samples were freeze‐dried, reconstituted in 0.1% formic acid, and subjected to analysis using an LTQ mass spectrometer (Thermo Finnigan, CA) connected to an Agilent 1200 capillary system (Agilent, Germany). Subsequently, the LTQ mass spectrometer with the nano‐ESI source was used to identify the eluted peptide ions. For MS/MS, a normalized collision energy of 35% was utilized along with an electrospray voltage of 1.9 kV. Data‐dependent MS/MS spectra were obtained in which the five most abundant spectra from the full MS scan were selected for fragmentation. The Dynamic Exclusion settings included a repeat count of 1, a repeat duration of 30 s, a dynamic exclusion duration of 180 s, an exclusion mass width of 1.5 Da, and a list of 50 dynamic exclusions.

### 2.11 Statistical analysis

GraphPad Prism 9.0 was utilized for statistical analyses, with three or five independent replicates conducted for all experiments. Mean ± standard deviation is used to present the data in all figures. Group differences were assessed using the appropriate statistical tests, including the Student's *t*‐test, one‐way analysis of variance (ANOVA), or two‐factor ANOVA. A *p*‐value below 0.05 (two‐tailed) was deemed to have statistical significance.

## 3 RESULTS

### 3.1 Characterization of BMSCs and BMSC‐MitoEVs

**FIGURE 1:**
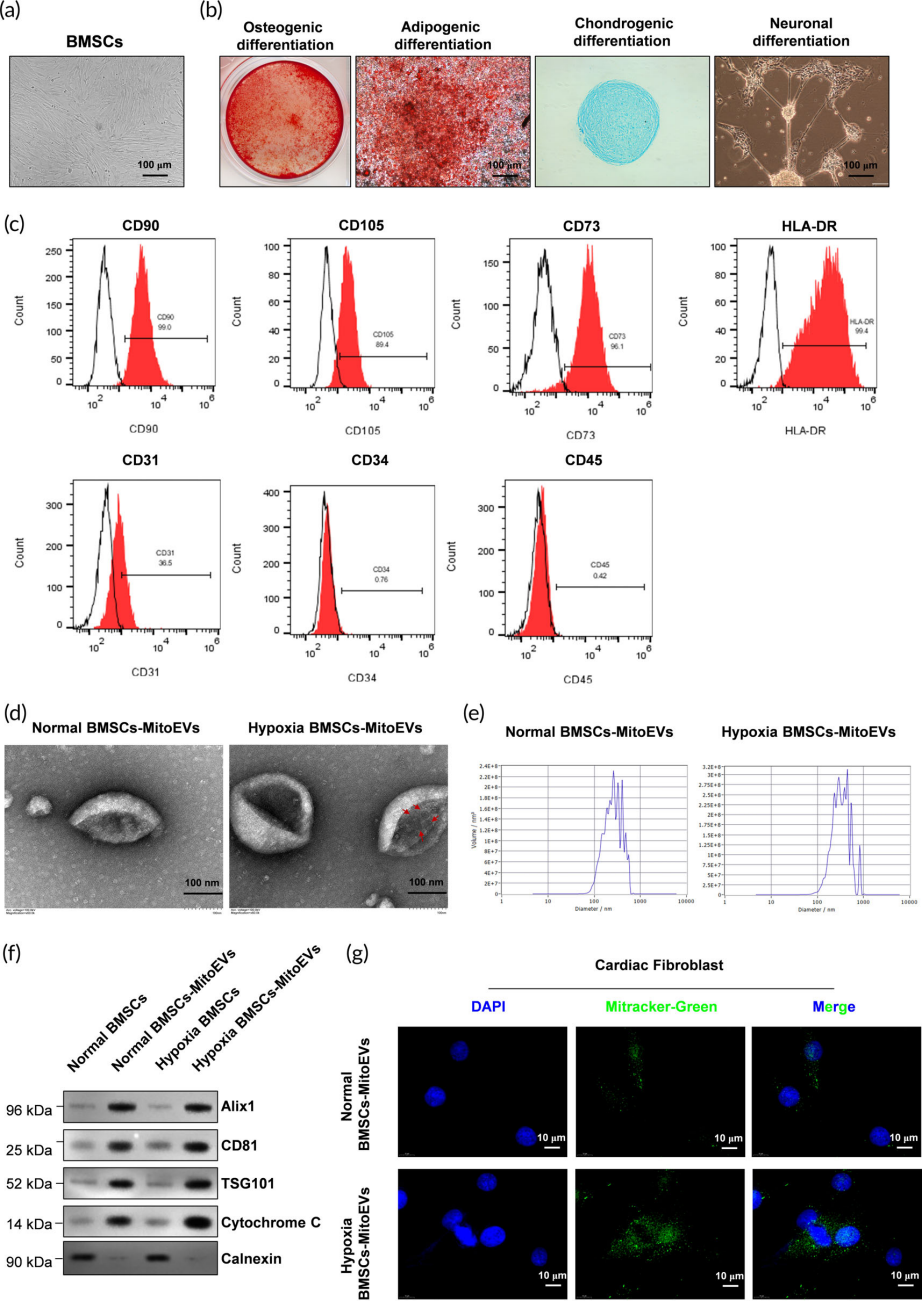
Characterization of BMSCs and BMSC‐derived mitochondria‐containing extracellular vesicles (MitoEVs). (a) BMSCs exhibit a spindle‐like morphology. (b) BMSCs differentiate into osteoblasts, adipocytes, chondroblasts, and neuron‐like cells under lineage‐specific induction. (c) Flow cytometry confirms expression of mesenchymal markers (CD29, CD44, and CD90) and absence of hematopoietic markers (CD34 and CD45). (d) Transmission electron microscopy (TEM) reveals exosome‐like vesicles (scale bar: 100 nm). Hypoxia BMSC‐MitoEVs contain mitochondria with intact cristae (arrows). (e) Nanoparticle tracking (Nanosight) shows BMSC‐EV size distribution (peak: 110 nm). (f) Western blot confirms EV markers (Alix1, CD81, and TSG101) and mitochondrial cytochrome c in Hypoxia MitoEVs. Calnexin (ER marker) is absent. (g) MitoTracker Green‐labeled Hypoxia MitoEVs (green) are internalized by cardiac fibroblasts (DAPI: Blue nuclei; scale bar: 10 μm). *N* = 3.

Primary BMSCs exhibited a spindle‐shaped morphology (Figure 1a) and demonstrated multipotent differentiation potential, successfully differentiating into osteoblasts, adipocytes, chondroblasts, and neuron‐like cells under lineage‐specific induction protocols (Figure 1b). Flow cytometry confirmed the expression of mesenchymal surface markers (CD73, CD90, CD105) and the absence of hematopoietic markers (CD45, CD19, CD14), validating BMSC purity (Figure 1c). TEM revealed EVs isolated from BMSC‐conditioned media with a cup‐shaped morphology typical of exosomes (~100 nm diameter; Figure 1d). Hypoxia‐preconditioned BMSC‐derived MitoEVs (Hypoxia MitoEVs) contained intact mitochondrial structures, identifiable by double membranes and cristae (Figure 1d, arrowheads). Nanoparticle tracking analysis (Nanosight) confirmed a homogeneous EV size distribution (peak: 110 nm; Figure 1e). Western blot analysis showed positive expression of EV markers (Alix1, CD81, TSG101) and mitochondrial cytochrome c in Hypoxia MitoEVs, while the endoplasmic reticulum marker calnexin was absent (Figure 1f). Functional uptake assays demonstrated that MitoTracker Green‐labeled Hypoxia MitoEVs were internalized by CFs and localized to the perinuclear region within 24 h(Figure 1g).

### 3.2 MitoEVs derived from hypoxia preconditioned‐BMSCs significantly inhibit the TGF‐*β*1‐induced myofibroblast transformation of CFs in vitro

**FIGURE 2:**
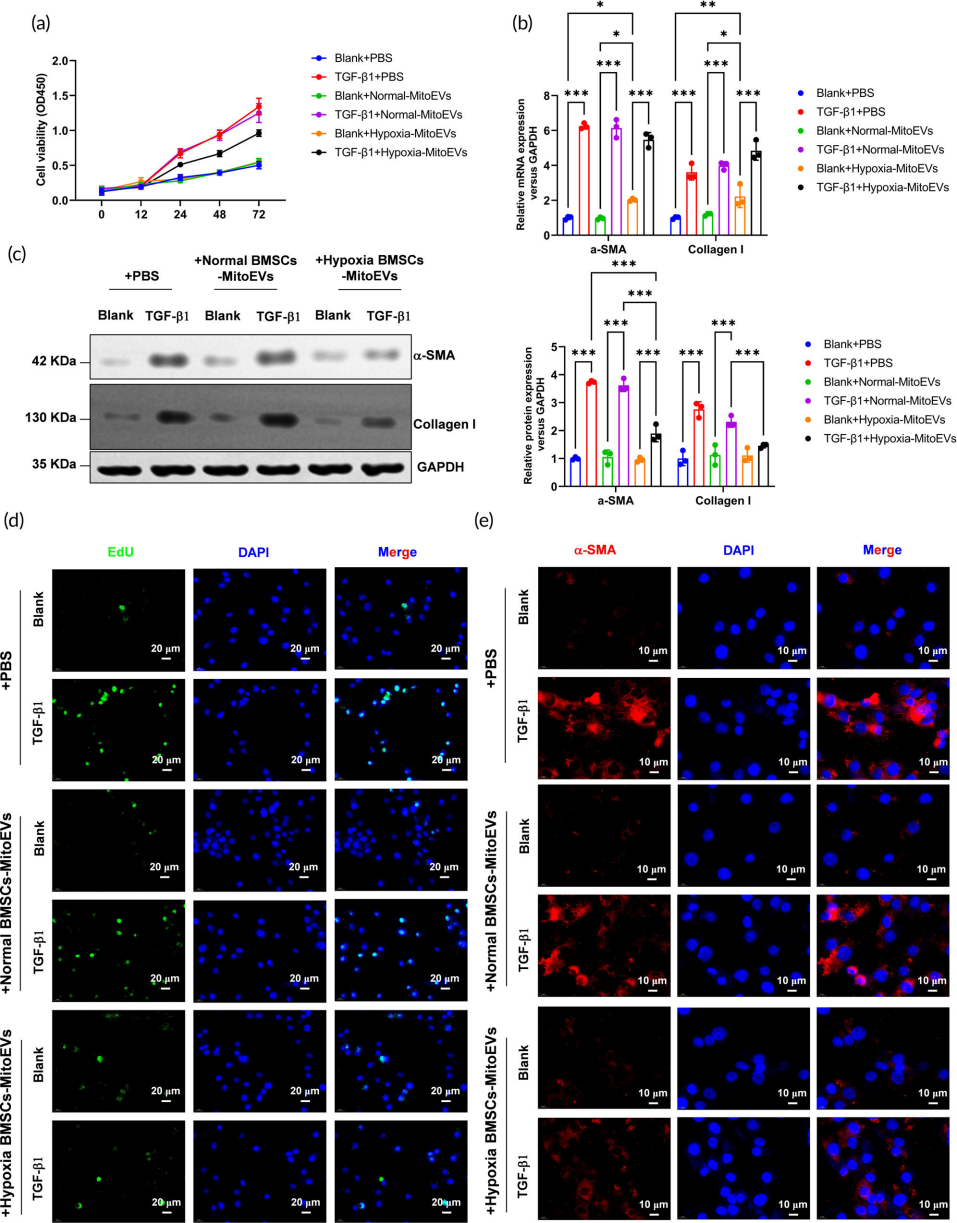
Hypoxia‐preconditioned BMSC‐MitoEVs suppress TGF‐β1‐induced myofibroblast transformation in cardiac fibroblasts. (a) TGF‐*β*1 (10 ng/mL, 24 h) increases CF proliferation (CCK‐8 assay), which is reversed by Hypoxia MitoEVs (50 μg/mL). (b) TGF‐*β*1 elevates *α*‐SMA and Col1a1 mRNA levels (qRT‐PCR), attenuated by Hypoxia MitoEVs. (c) Hypoxia MitoEVs reduce TGF‐*β*1‐induced *α*‐SMA and Col1a1 protein expression (Western blot). (d) EdU assay confirms reduced CF proliferation with Hypoxia MitoEVs (scale bar: 20 μm). (e) Immunofluorescence shows Hypoxia MitoEVs diminish *α*‐SMA+ stress fibers (scale bar: 10 μm). *N* = 3; *p* < 0.05, **p*< 0.01, **p < 0.001 versus indicated groups.

Transforming growth factor‐*β*1 (TGF‐*β*1; 10 ng/mL, 24 h) induced CF activation, marked by increased proliferation (CCK‐8 assay; Figure 2a) and elevated expression of myofibroblast markers α‐smooth muscle actin (*α*‐SMA) and collagen type I alpha 1 (Col1a1) at mRNA (Figure 2b) and protein levels (Figure 2c). Hypoxia MitoEV treatment (50 μg/mL) significantly attenuated these effects, reducing proliferation (Figure 2a) and suppressing *α*‐SMA/Col1a1 expression by 60%–70% (Figure 2b, c). EdU incorporation assays confirmed a 55% reduction in proliferating CFs (Figure 2d), while immunofluorescence revealed diminished *α*‐SMA+ stress fibers (Figure 2e). These findings demonstrate that Hypoxia MitoEVs inhibit TGF‐*β*1‐driven myofibroblast differentiation.

### 3.3 MitoEVs derived from hypoxia preconditioned‐BMSCs significantly attenuated the MI‐induced cardiac fibrosis

**FIGURE 3:**
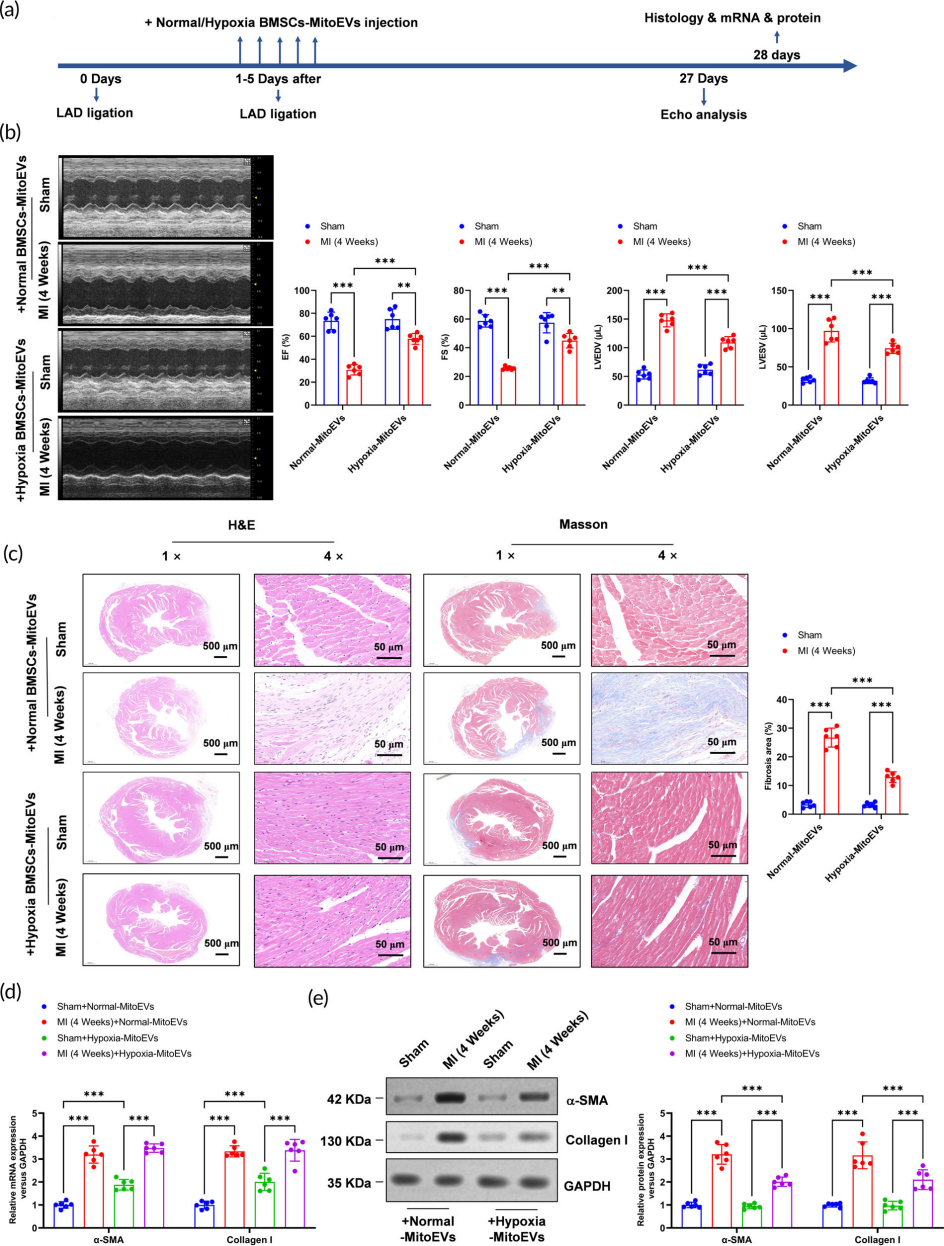
Hypoxia‐preconditioned BMSC‐MitoEVs attenuate post‐MI cardiac fibrosis in vivo. (a) Timeline: MitoEVs (100 μg/mouse) injected 24 h post‐LAD ligation; assessments at 4 weeks. (b) Echocardiography shows Hypoxia MitoEVs improve LVEF, LVFS, and LV volumes (LVESV and LVEDV). (c) H&E and Masson's trichrome staining reveal reduced infarct size and fibrosis with Hypoxia MitoEVs. (d) Hypoxia MitoEVs increase *α*‐SMA and Col1a1 mRNA in sham hearts but not post‐MI. (e) Hypoxia MitoEVs suppress *α*‐SMA and Collagen I protein levels post‐MI. *N* = 6; *p* < 0.05, **p*< 0.01, **p < 0.001 versus indicated groups.

Hypoxia MitoEVs (100 μg/mouse) administered intravenously 24 h post‐MI (LAD ligation), the timeline was shown in Figure 3a. Hypoxia MitoEVs improved cardiac function at 4 weeks, as shown by increased left ventricular ejection fraction (LVEF, Figure 3b) and fractional shortening (LVFS, Figure 3b), alongside reduced LV end‐systolic volume (LVESV, Figure 3b). Histological analysis revealed reduced infarct size (H&E, Figure 3c) and collagen deposition (Masson's trichrome staining, Figure 3c). While *α*‐SMA/Col1a1 mRNA levels remained unchanged in sham hearts (Figure 3d), Hypoxia MitoEVs suppressed their protein expression post‐MI (Figure 3e), confirming antifibrotic efficacy.

### 3.4 MitoEVs derived from hypoxia preconditioned‐BMSCs significantly attenuated TGF‐*β*1 impaired mitochondrial oxidative phosphorylation and elevated glycolysis in CF

**FIGURE 4:**
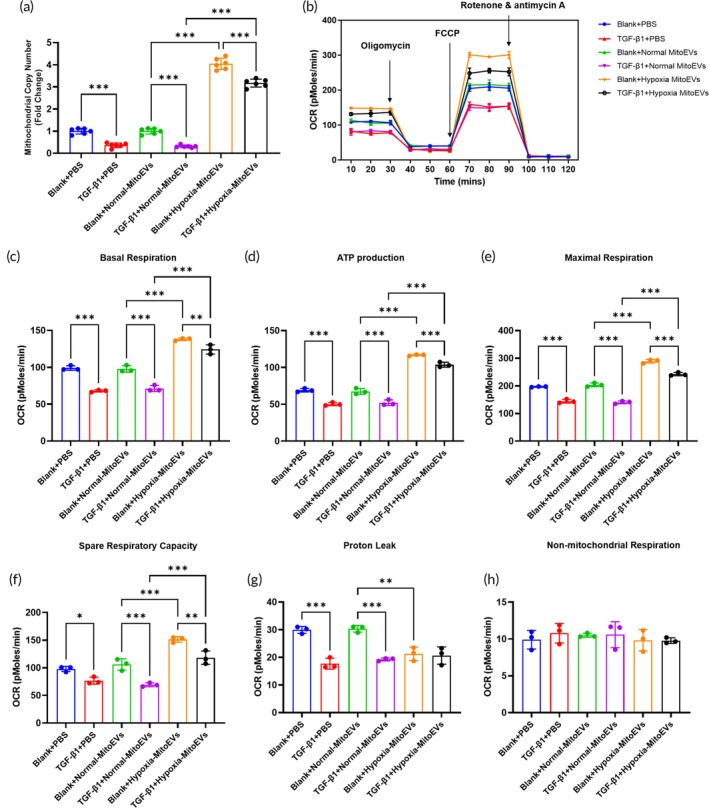
Hypoxia‐preconditioned BMSC‐MitoEVs restore mitochondrial oxidative phosphorylation in TGF‐*β*1‐treated CFs. (a) TGF‐*β*1 reduces mitochondrial number, reversed by Hypoxia MitoEVs. (b) Seahorse XF24 analysis: OCR measured after sequential oligomycin, FCCP, and rotenone/antimycin A injections. (c)–(f) Hypoxia MitoEVs rescue basal OCR, ATP production, maximal respiration, and spare respiratory capacity. (e), (f) Proton leak and non‐mitochondrial respiration are normalized. *N* = 3; *p* < 0.05, **p*< 0.01, **p < 0.001 versus indicated groups.

**FIGURE 5:**
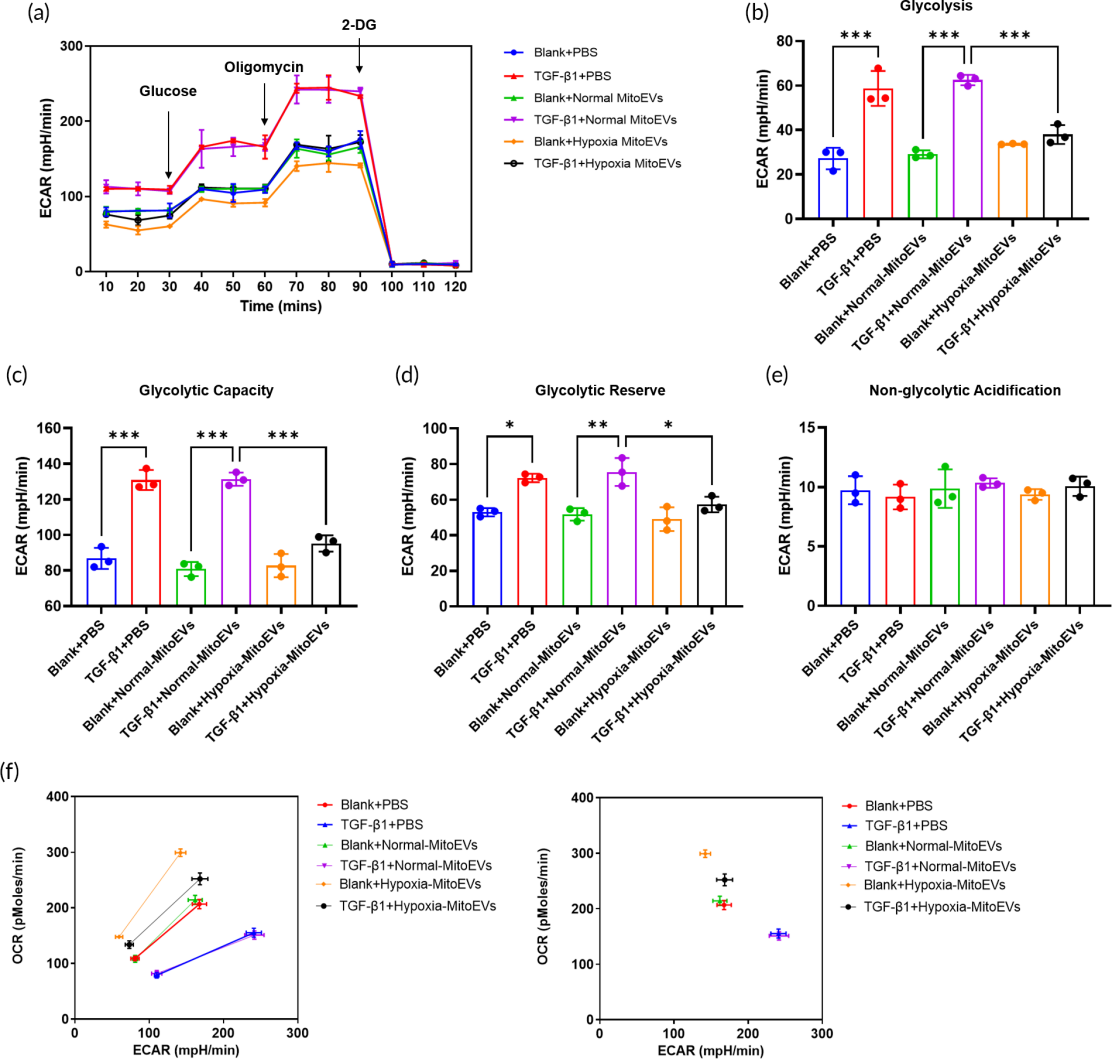
Hypoxia‐preconditioned BMSC‐MitoEVs suppress TGF‐*β*1‐induced glycolysis in CFs. (a) Seahorse extracellular acidification rate (ECAR) measurements. (b)–(d) Hypoxia MitoEVs reduce basal glycolysis, glycolytic capacity, and reserve. (E) Non‐glycolytic acidification is unaffected. (f) OCR/ECAR ratio is restored to metabolic balance. *N* = 3; *p* < 0.05, **p*< 0.01, **p < 0.001 versus indicated groups.

TGF‐*β*1 reduced mitochondrial copy number in CFs by 40% (Figure 4a), while Hypoxia MitoEVs restored mitochondrial content (Figure 4a). Seahorse XF24 analysis (Figure 4b) showed Hypoxia MitoEVs rescued TGF‐β1‐impaired mitochondrial respiration, increasing basal OCR (Figure 4c), ATP production (Figure 4d), maximal respiration (Figure 4e), and spare respiratory capacity (Figure 4f). The proton leak (Figure 4g) and non‐mitochondrial respiration (Figure 4h) were unchanged with the Hypoxia MitoEVs treatment. Conversely, Hypoxia MitoEVs impaired TGF‐*β*1 elevated glycolysis (ECAR, Figure 5a), including decreasing basal glycolysis (Figure 5b), glycolytic capacity (Figure 5c), and glycolytic reserve (Figure 5d). The non‐glycolytic acidification (Figure 5e) was unchanged with the Hypoxia MitoEVs treatment. The OCR/ECAR ratio (Figure 5f) shifted from ECAR to OCR, indicating restored metabolic balance.

### 3.5 Inhibition of mitochondrial ATP synthase in MitoEVs from hypoxia preconditioned BMSCs fails to inhibit the TGF‐β1‐induced myofibroblast transformation of CFs in vitro

**FIGURE 6:**
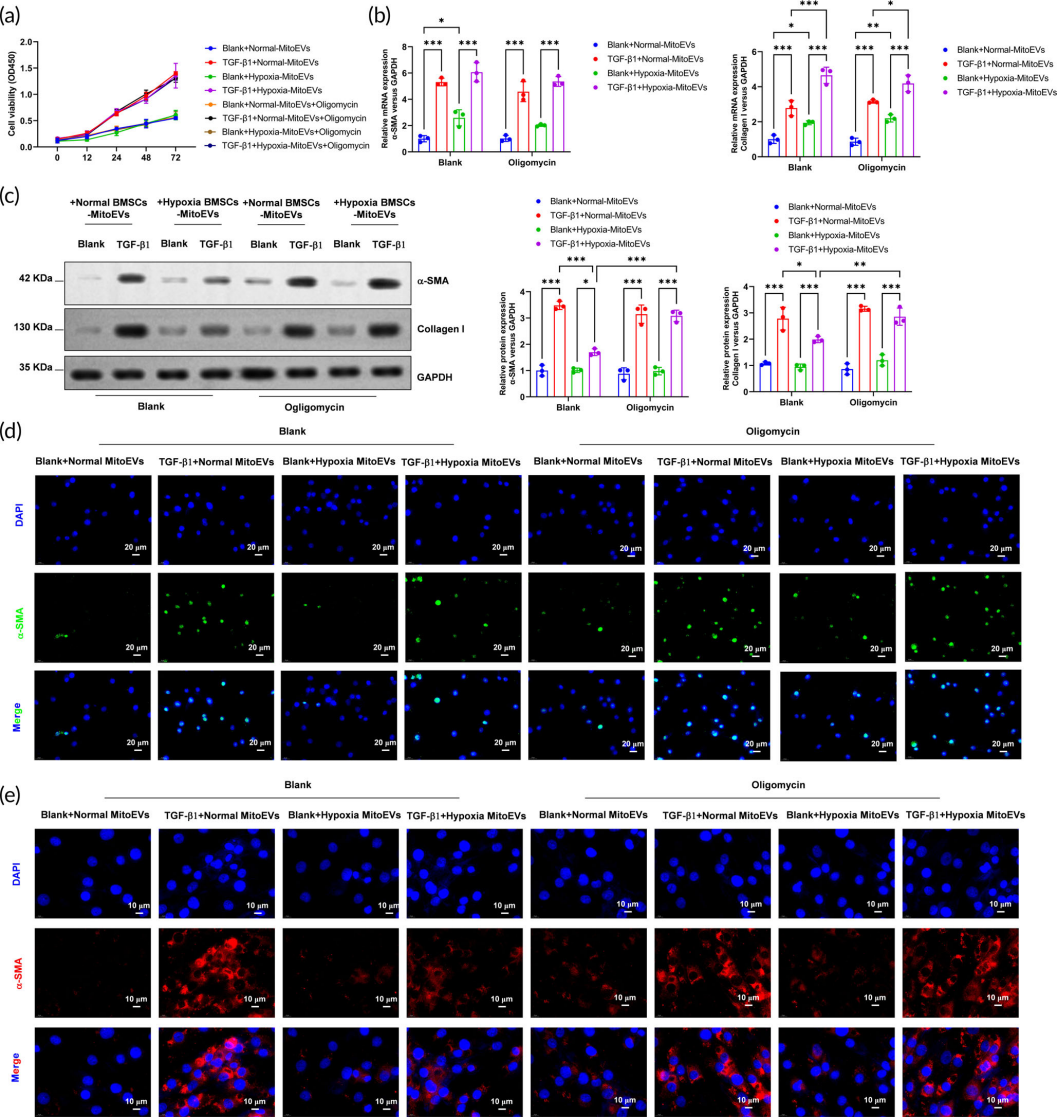
Mitochondrial ATP synthase inhibition abolishes Hypoxia MitoEVs' antifibrotic effects in vitro. (a) Oligomycin (1 μM) does not alter CF proliferation (CCK‐8). (b) Hypoxia MitoEVs fail to suppress α‐SMA/Col1a1 mRNA under oligomycin. (c) Oligomycin reverses Hypoxia MitoEV‐mediated *α*‐SMA/Col1a1 protein reduction. (d) EdU assay confirms loss of antiproliferative effects (scale bar: 20 μm). (e) *α*‐SMA immunofluorescence shows oligomycin restores stress fibers (scale bar: 10 μm). *N* = 3; *p* < 0.05, **p*< 0.01, **p < 0.001 versus indicated groups.

To validate the functional contribution of mitochondrial components in MitoEVs from hypoxia‐preconditioned BMSCs (Hypoxia‐BMSC MitoEVs) during myofibroblast transformation, we inhibited mitochondrial ATP synthase with oligomycin in MitoEVs isolated from normoxic or hypoxic BMSCs. Unexpectedly, oligomycin treatment reversed the Hypoxia‐BMSC MitoEV‐mediated suppression of CF proliferation (Figure 6a). While mRNA levels of α‐SMA and Col1a1 remained unaffected by oligomycin (Figure 6b), protein expression of these fibrotic markers—which was initially reduced by Hypoxia‐BMSC MitoEVs in TGF‐β1‐treated cells—was restored following ATP synthase inhibition (Figure 6c). EdU incorporation assays (Figure 6d) and immunofluorescence (Figure 6e) confirmed increased proliferation and *α*‐SMA/Col1a1 protein levels, respectively. These findings demonstrate that blocking mitochondrial ATP synthase activity in Hypoxia‐BMSC MitoEVs fails to abrogate their pro‐fibrotic gene translation‐suppressing effects in vitro.

### 3.6 Inhibition of mitochondrial ATP synthase in MitoEVs from hypoxia‐preconditioned BMSCs fails to attenuate the MI‐induced cardiac fibrosis

**FIGURE 7:**
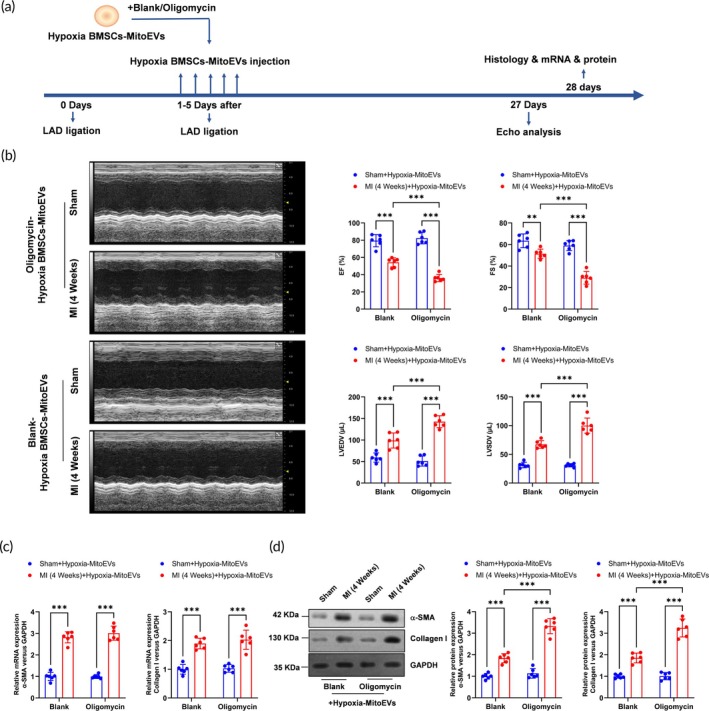
Mitochondrial ATP synthase inhibition negates hypoxia MitoEVs' therapeutic efficacy post‐MI. (a) Timeline: Oligomycin‐treated hypoxia MitoEVs injected post‐MI. (b) Echocardiography reveals worsened LVEF, LVFS, and LV volumes. (c) H&E and Masson's staining show increased infarct size and fibrosis. (d) *α*‐SMA/Collagen I protein levels remain elevated. *N* = 6; *p* < 0.05, **p*< 0.01, **p < 0.001 versus indicated groups.

To assess the in vivo relevance of mitochondrial ATP synthase activity in Hypoxia‐BMSC MitoEVs, we administered oligomycin‐treated MitoEVs to mice 1 day post‐MI, with echocardiography performed at 4 weeks (Figure 7a). Hypoxia‐BMSC MitoEVs significantly improved cardiac function post‐MI, as shown by elevated ejection fraction (EF) and fractional shortening (FS), alongside reduced left ventricular volumes (Figure 7b). Strikingly, oligomycin‐treated Hypoxia‐BMSC MitoEVs abolished these benefits, exacerbating contractile dysfunction. While fibrotic gene mRNAs remained unchanged (Figure 7c), protein levels of *α*‐SMA and Col1a1 increased in hearts receiving oligomycin‐treated MitoEVs (Figure 7d). These data reveal that mitochondrial ATP synthase inhibition in Hypoxia‐BMSC MitoEVs negates their anti‐fibrotic efficacy in vivo.

### 3.7 MitoEVs from hypoxia‐preconditioned BMSCs deliver QKI protein into cardiac fibrosis and restrict the mRNA translation of fibrotic gene's mRNA

**FIGURE 8:**
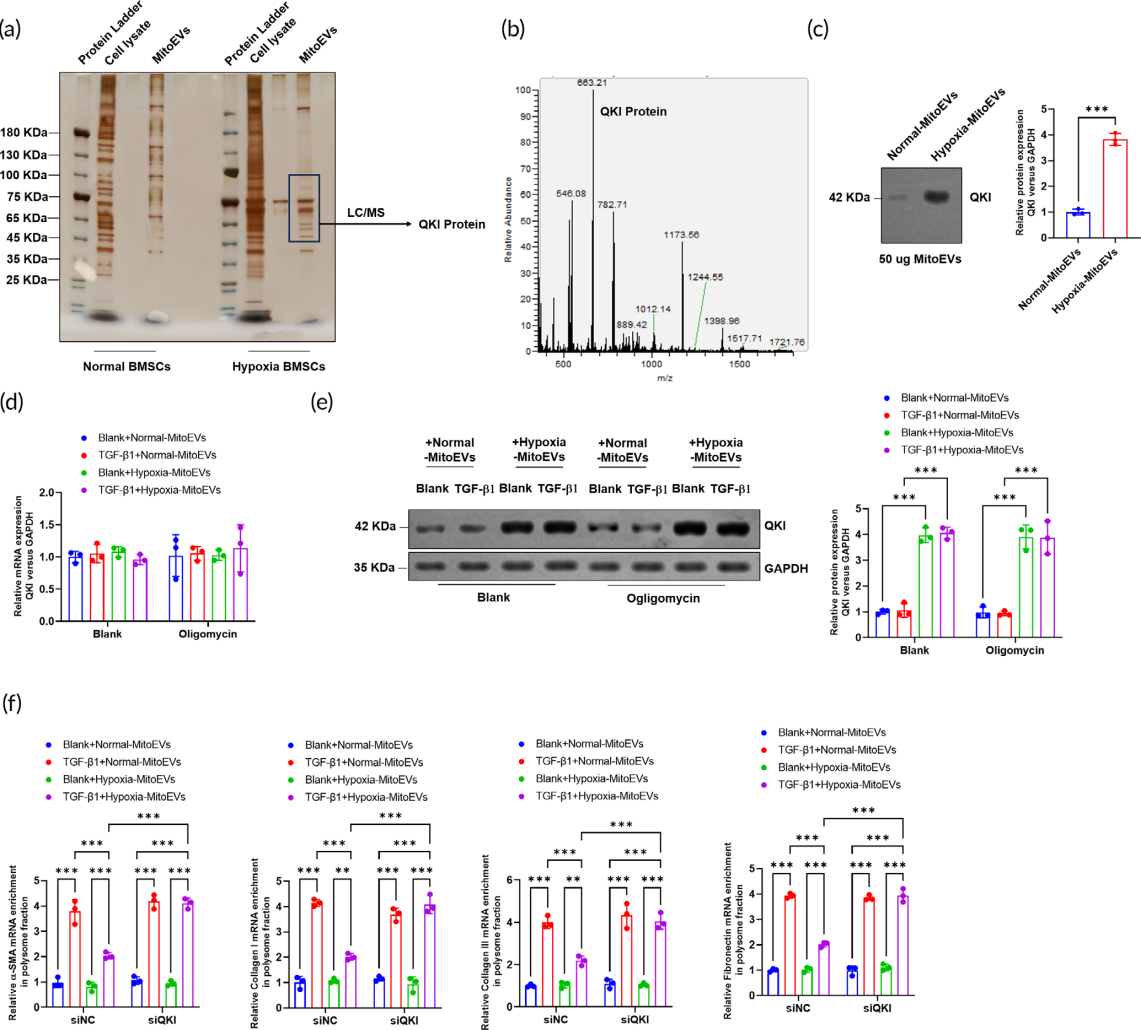
Hypoxia MitoEVs deliver QKI protein to suppress fibrotic mRNA translation. (a), (b) Silver stain and LC–MS/MS identify QKI as hypoxia‐upregulated in MitoEVs. (c) Western blot confirms QKI enrichment in hypoxia MitoEVs. (d) QKI mRNA in CFs is unchanged by hypoxia MitoEVs. (e) Hypoxia MitoEVs increase QKI protein in CFs. (f) Ribosome profiling shows QKI knockdown in hypoxia MitoEVs restores fibrotic mRNA translation. *N* = 3; *p* < 0.05, **p*< 0.01, **p < 0.001 versus indicated groups.

Proteomic analysis of MitoEVs from normoxic versus hypoxic BMSCs via SDS‐PAGE and LC–MS/MS (Figure 8a, b) identified Quaking (QKI), an m7G‐modified RNA‐binding protein, as a cargo enriched in Hypoxia‐BMSC MitoEVs (Figure 8c). Western blotting confirmed QKI transfer to CFs, with no changes in QKI mRNA (Figure 8d), indicating direct protein delivery (Figure 8e). QKI is known to interact with G3BP1 in stress granules (SGs), where it inhibits translation of m7G‐modified transcripts. Knocking down QKI in BMSCs prior to MitoEV isolation reversed the suppression of fibrotic gene (*α*‐SMA, collagens I/III, fibronectin) mRNA translation efficiency observed in recipient CFs (Figure 8f). These results establish that QKI delivery via Hypoxia‐BMSC MitoEVs restricts fibrotic protein synthesis at the translational level.

### 3.8 Knockdown of QKI in hypoxia‐preconditioned BMSC‐MitoEVs abrogates their therapeutic efficacy against cardiac fibrosis post‐MI

**FIGURE 9:**
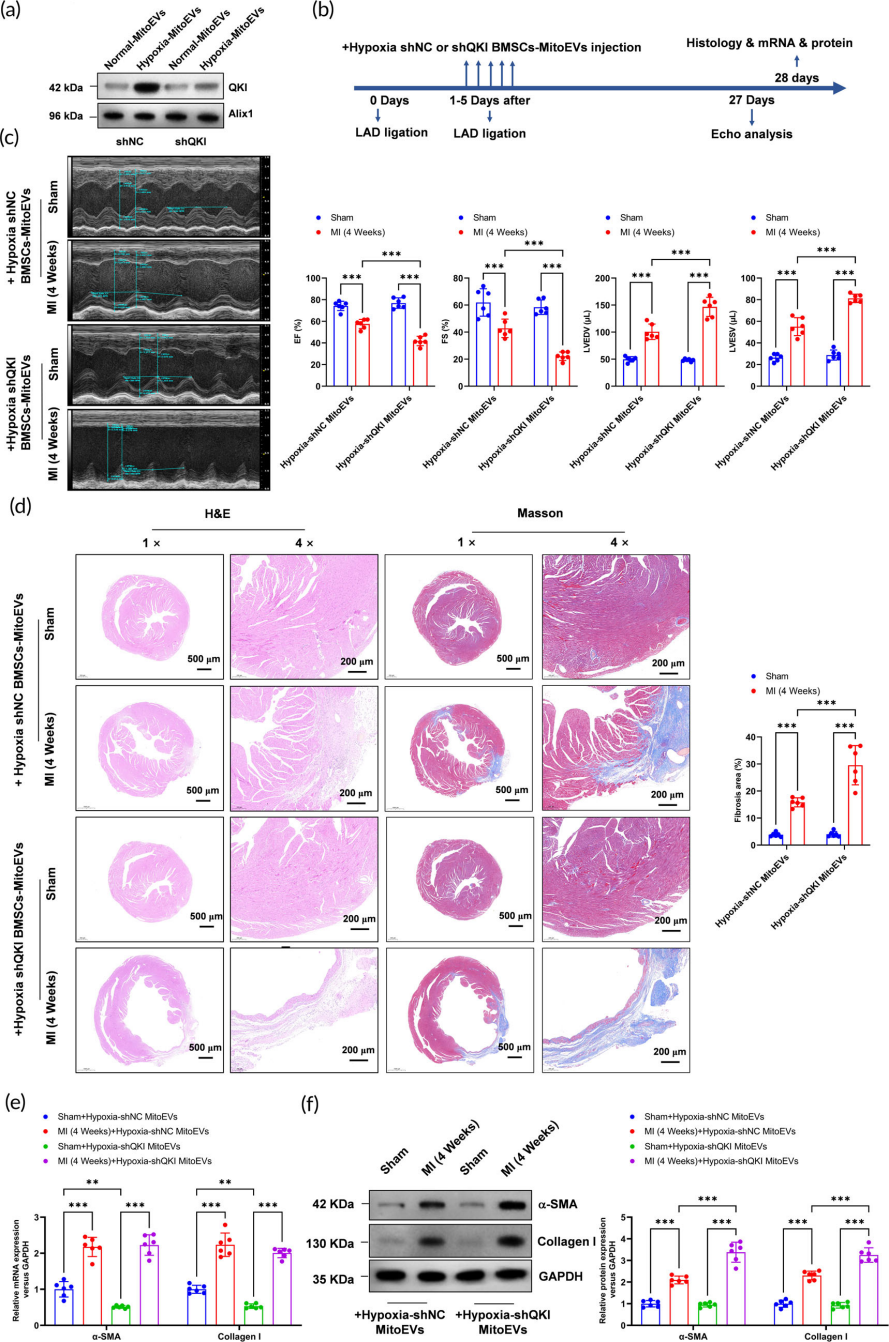
QKI knockdown in Hypoxia MitoEVs reduces their antifibrotic efficacy post‐MI. (a) QKI knockdown efficiency in Hypoxia MitoEVs (Western blot). (b) Timeline: ShQKI or shNC MitoEVs injected post‐MI. (c) Echocardiography shows worsened cardiac function with shQKI MitoEVs. (d) H&E and Masson's staining reveal increased injury and fibrosis. (e) shQKI MitoEVs reduce α‐SMA/Col1a1 mRNA in sham hearts but fail post‐MI. (f) *α*‐SMA/Collagen I protein levels remain elevated post‐MI. *N* = 6; *p* < 0.05, **p*< 0.01, **p < 0.001 versus indicated groups.

To elucidate the functional role of the RNA‐binding protein QKI delivered by hypoxia‐preconditioned BMSC‐MitoEVs, we genetically silenced QKI in BMSCs using shRNA (shQKI) prior to MitoEV isolation. Western blot analysis confirmed a 70% reduction in QKI protein levels in shQKI MitoEVs compared to scrambled control (shNC) MitoEVs (Figure 9a). In vivo, shQKI or shNC MitoEVs (100 μg/mouse) were administered intravenously 24 h post‐MI, with echocardiographic and histological assessments performed at 4 weeks (Figure 9b). Strikingly, shQKI MitoEVs failed to improve cardiac function, as evidenced by significantly reduced LVEF (Figure 9b) and LVFS (Figure 9b), alongside increased LVESV (Figure 9b). Histological analysis revealed exacerbated tissue injury in the shQKI‐MitoEV group, with larger infarct areas (H&E) and increased collagen deposition (Masson's fibrosis area, Figure 9c). Intriguingly, while shQKI‐MitoEVs reduced α‐SMA and Col1a1 mRNA levels in sham‐operated hearts (Figure 9d), this effect was lost post‐MI, with no significant differences in fibrotic mRNA levels between shQKI‐ and shNC‐MitoEV‐treated MI hearts (Figure 9d). Conversely, QKI knockdown abolished the MitoEV‐mediated suppression of fibrotic protein expression in infarcted hearts, elevating *α*‐SMA and Collagen I (Figure 9e). These findings demonstrate that QKI cargo in hypoxia‐preconditioned MitoEVs is indispensable for their antifibrotic efficacy, likely by regulating the translational machinery of profibrotic genes in CFs during post‐MI remodeling.

## 4 DISCUSSION

Cardiac fibrosis is a common complication following MI, contributing to impaired cardiac function and the development of heart failure. Traditional treatment options for cardiac fibrosis are limited, necessitating the exploration of innovative strategies.^10^, ^11^ This study investigates the use of MiotEVs derived from hypoxia‐preconditioned BMSCs as a novel therapeutic approach. The study highlights the regenerative and anti‐fibrotic properties of these vesicles and emphasizes the significance of hypoxic preconditioning in enhancing their therapeutic efficacy.

In several preclinical and clinical studies, MSCs and their EVs have shown promise in treating various diseases, including cardiovascular diseases, neurological disorders, autoimmune diseases, and tissue injuries.^14^, ^17^, ^19^ For example, in cardiovascular diseases, MSCs and their EVs have demonstrated the ability to improve cardiac function, neovascularization, and reduce fibrosis through paracrine signaling and modulation of immune responses. Similarly, in neurological disorders, MSCs and their EVs have shown neuroprotective and regenerative effects through the secretion of trophic factors and the promotion of neural cell survival and differentiation. The disparity in oxygen concentration between in vivo and in vitro is widely recognized. Typically, cell culture in a laboratory setting necessitates the presence of 21% oxygen, whereas in living organisms, MSCs are found in an environment with oxygen levels around 5%.^24^ Moreover, research has validated that hypoxia has the ability to alter the physiological mechanisms of MSCs. Newly available data indicates that various mitochondrial contents are highly concentrated in specific EV subgroups, and these mitoEVs have the ability to transport mitochondrial components and influence the operations of recipient cells in various circumstances, which has become a prominent subject of interest within this domain. However, the role of MitoEVs from hypoxia‐preconditioned BMSCs in cardiac fibrosis remains unclear. In this study, the authors investigated the potential therapeutic effects of MitoEVs derived from hypoxia‐preconditioned BMSCs in cardiac fibrosis. They evaluated the impact of MitoEVs on the myofibroblast transformation of CFs in vitro and assessed their therapeutic potential in a MI mouse model. The results of their in vitro experiments demonstrated that MitoEVs derived from hypoxia‐preconditioned BMSCs significantly inhibited the transformation of CFs into myofibroblasts induced by TGF‐*β*1. This was evidenced by the inhibition of proliferation and downregulation of fibrosis‐related markers such as *α*‐SMA and Col1a1. These findings suggest that MitoEVs derived from hypoxia‐preconditioned BMSCs have anti‐fibrotic properties and can prevent the pathological transformation of CFs into myofibroblasts. To further validate their findings in vivo, the researchers treated MI mice with MitoEVs derived from normal or hypoxia‐preconditioned BMSCs. The results showed that MitoEVs derived from hypoxia‐preconditioned BMSCs significantly improved cardiac function, as indicated by increased EF, FD, and decreased left ventricular end‐diastolic volume and left ventricular end‐systolic volume. Histological analysis also revealed reduced structural damage and collagen accumulation in the left ventricular tissues of MI mice treated with hypoxia‐preconditioned BMSC‐derived MitoEVs.

Reprogramming of energy metabolism has emerged as a key mechanism in cardiac fibrosis, a pathological process characterized by an excessive accumulation of ECM in the heart. The shift in energy metabolism from oxidative phosphorylation (OXPHOS) to glycolysis and fatty acid oxidation (FAO) contributes to the pathological remodeling of cardiac tissue and the development of fibrosis.^26^ Glycolysis is a less efficient form of energy production compared to OXPHOS, but it provides cells with a rapid source of energy and metabolic intermediates.^27^ In cardiac fibrosis, the upregulation of glycolytic enzymes such as hexokinase and lactate dehydrogenase has been reported in fibroblasts and myofibroblasts.^28^ This promotes cell proliferation and migration, leading to the accumulation of ECM.^29^ In this study, we found that MitoEVs from hypoxia‐preconditioned BMSCs may have potential therapeutic effects on cardiac fibrosis. The study investigated the effect of MitoEVs on mitochondrial function and glycolysis in TGF‐*β*1‐treated CFs. The results showed that TGF‐*β*1 impaired mitochondrial oxidative phosphorylation and increased glycolysis in CFs, which are both associated with cardiac fibrosis. However, MitoEVs from hypoxia‐preconditioned BMSCs reversed these effects by increasing mitochondrial copy number and improving mitochondrial respiration, while decreasing glycolysis.

RNA m7G, also known as 7‐methylguanosine, is a modified nucleotide commonly found at the 5′ end of eukaryotic mRNA molecules.^30^ It plays a critical role in various cellular processes, including mRNA stability, translation initiation, and RNA splicing.^25^ Dysregulation of m7G modification has been associated with several diseases, highlighting its significance in maintaining cellular homeostasis.^31^, ^32^, ^33^, ^34^ We previously reported that fibroblast‐specific knockout of METTL1 attenuates MI‐induced cardiac fibrosis.^32^ According to the report, QKI7 engages with the SG core protein G3BP1 through its C terminus and transports internally modified m7G transcripts into SGs to control mRNA stability and translation during stressful situations. To make cancer cells more responsive to chemotherapy, QKI7 reduces the translation efficiency of crucial genes in the Hippo signaling pathways.^35^ In this study, we identified that MitoEVs from hypoxia‐preconditioned BMSCs were enriched with QKI protein. Strikingly, MitoEVs from hypoxia‐preconditioned BMSCs delivered the QKI protein into CFs and restricted the mRNA translation of fibrotic gene's mRNA under fibrosis activation.

**FIGURE 10:**
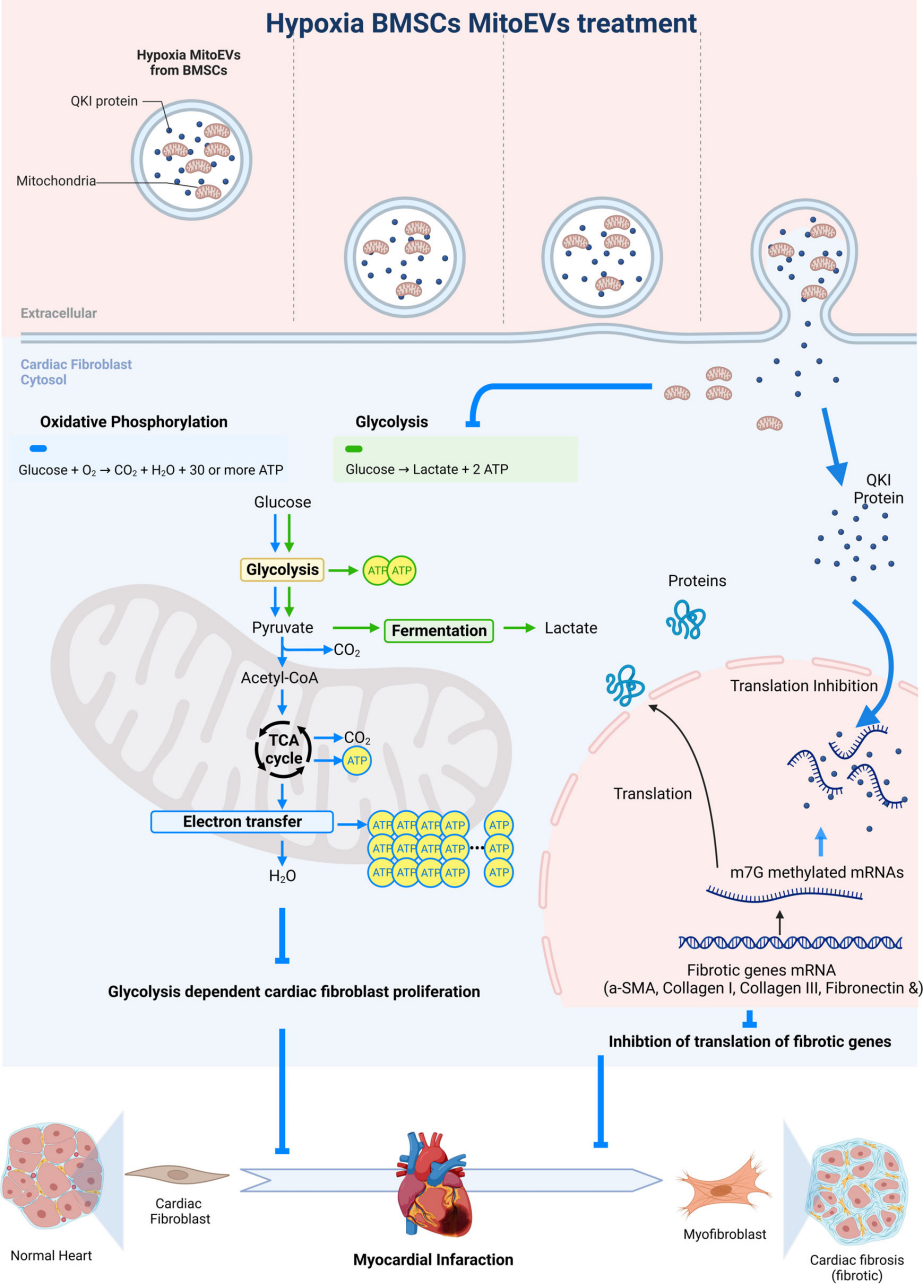
Schematic: Hypoxia‐preconditioned BMSC‐MitoEVs attenuate cardiac fibrosis via dual mechanisms. Hypoxia MitoEVs deliver functional mitochondria to restore oxidative phosphorylation (OXPHOS) and transfer QKI protein to inhibit fibrotic mRNA translation in activated cardiac fibroblasts.

Collectively, the findings of this study demonstrate that MitoEVs from hypoxia‐preconditioned MitoEVs show promising anti‐fibrotic effects. On one hand, MitoEVs deliver healthy mitochondria to reverse the energy metabolism reprogram from glycolysis to OXPHOS in activated CFs. On the other hand, MitoEVs deliver QIK protein to restrict the mRNA translation of fibrotic genes' mRNA under fibrosis activation (Figure 10). These positive outcomes suggest that hypoxia‐preconditioned MitoEVs derived from BMSCs have the potential to effectively address the detrimental effects of cardiac fibrosis.

## AUTHOR CONTRIBUTIONS

Liang Wang designed this study. Jungang Nie and Liang Wang performed all experiments; Hongwen Zhu and Zhiming Gao analyzed and interpreted the data; Liang Wang drafted the manuscript; Jungang Nie revised the manuscript. All authors read and approved the final manuscript.

## FUNDING INFORMATION

This work was supported by grants from the Jiangxi Province Science and Technology Department project (No. 20224BAB206011 and No. 20232BAB206015) and the Technology Research Project of Jiangxi Provincial Department of Education (No. GJJ2200145).

## CONFLICT OF INTEREST STATEMENT

The authors declare no conflict of interest.

## CONSENT FOR PUBLICATION

All the authors have consented to the publication of this research.

## Supporting information


**Supplementary Figure 1.** The protocol of the isolation of MitoEV from normal and hypoxia‐preconditioned MiotEVs from bone marrow mesenchymal stem cells.

**Supplementary Table 1.** The sequence of the primers used in this study.

## Data Availability

The data that support the findings of this study are available from the corresponding author upon reasonable request.
