# Supplementary material for: Hypoxia‐preconditioned MiotEVs from bone marrow mesenchymal stem cells inhibit myocardial infarction‐induced cardiac fibrosis
Source: Bioeng Transl Med. 2025 Jul 4;11(1):e70046. doi: 10.1002/btm2.70046 (PMC12821231; doi:10.1002/btm2.70046)
Supplement: Supplementary file 2 — Supplementary Table 1. The sequence of the primers used in this study. [file BTM2-11-e70046-s002.docx]

**Hypoxia-preconditioned MiotEVs from bone marrow mesenchymal stem cells inhibit myocardial infarction-induced cardiac fibrosis**

Jungang Nie^1,2^, Hongwen Zhu^1,2^, Zhiming Gao^1,2^, Liang Wang^1,2*^

^1^Department of Cardiology, The First Affiliated Hospital of Nanchang University, Nanchang, Jiangxi, 330006, China.

^2^Hypertension Research Institute of Jiangxi Province, Nanchang, Jiangxi, 330006, China.

*Correspondence to: Dr. Liang Wang, Department of Cardiology, The First Affiliated Hospital of Nanchang University, Hypertension Research Institute of Jiangxi Province, No. 17 Yongwaizheng Street, Nanchang, Jiangxi, 330006, China. Email address: ndyfy02336@ncu.edu.cn

**Supplementary Table 1.** The sequence of the primers used in this study.

| **Genes** | **Forward primer (5’-3’)** | **Reverse primer (5’-3’)** |
| --- | --- | --- |
| ACTA2 | CCCAGACATCAGGGAGTAATGG | TCTATCGGATACTTCAGCGTCA |
| COL1A1 | GCTCCTCTTAGGGGCCACT | ATTGGGGACCCTTAGGCCAT |
| QKI | AGCAGCCTGCCCAACTTCTG | CGCGTCAGGCAATTCTGCAC |
| GAPDH | AGGTCGGTGTGAACGGATTTG | GGGGTCGTTGATGGCAACA |
